# Turning the tables on industry narratives: a health communication playbook for promoting health

**DOI:** 10.1093/heapro/daag130

**Published:** 2026-09-07

**Authors:** David Stuckler, Florence Berteletti, Martin McKee

**Affiliations:** Department of Social and Political Sciences, Bocconi University, Via Sarfatti, 10, Milan 20136, Italy; Independent Public Health Consultant, 115 Boulevard Guillaume Van Haelen, Forest 1190, Brussels, Belgium; Faculty of Public Health and Policy, London School of Hygiene & Tropical Medicine, 15-17 Tavistock Place, London WC1H 9SH, United Kingdom

**Keywords:** health communication, commercial determinants of health, public health advocacy, misinformation, policy influence, risk communication, health promotion strategy

## Abstract

Industries producing harmful products such as tobacco, alcohol, ultra-processed foods, and fossil fuels use highly developed communication strategies to shape public opinion, influence policy, and protect their markets. These ‘playbooks’ rely on recurring tactics, including framing harms as matters of personal choice, promoting innovation narratives, manufacturing doubt, delaying regulation, and obscuring human consequences. While public health has increasingly recognized these strategies, responses have often been fragmented and reactive. This paper argues that health promotion requires a practical communication playbook that complements existing strategic approaches by focusing on real-time tactical responses. Drawing on cross-sector evidence and the authors’ experience in public health policy and advocacy, we identify six core tactics: reframing choice as freedom from manipulation; shifting attention from innovation to accountability; pre-emptive prebunking of misleading narratives; amplifying scientific consensus while explaining uncertainty; responding with speed, unity, and accessible tools; and humanizing harms through stories and visuals. These tactics are supported by policy and system-level measures, including stronger regulatory safeguards, systematic monitoring of industry narratives, and coordinated cross-border infrastructure for intelligence and rapid response. Together, these elements form a practical framework for moving public health from reactive rebuttal to proactive narrative leadership. The paper highlights the need for greater preparedness, coordination, and investment in communication capacity, arguing that effective health promotion depends not only on evidence and policy but also on the ability to shape and sustain persuasive public narratives.

Contribution to health promotion

- Health promotion efforts have often focused on countering individual behaviours, overlooking how industry narratives shape choices and environments.
- This paper identifies common communication tactics used by harmful industries across sectors.
- It proposes a practical, action-oriented playbook to support proactive, coordinated public health responses.
- The framework highlights the importance of communication capacity, monitoring systems, and policy alignment.
- It offers actionable guidance for practitioners, researchers, and policy-makers to strengthen health promotion in complex commercial environments.

## Introduction

Industries that profit from harmful products, such as tobacco, alcohol, ultra-processed foods, and fossil fuels, have developed sophisticated ‘playbooks’ to shape public opinion, influence policy, and protect their markets (Reed *et al*. 2021, Moodie 2022, Thacker 2022). consumption as a matter of personal choice, presenting products as innovation, selectively deploying evidence, and obscuring risks. Together, these approaches enable corporate actors to maintain legitimacy while deflecting attention from population-level harms and structural drivers of ill health.

For health promotion, this presents a fundamental challenge. Efforts to improve health are often grounded in evidence, regulation, and behaviour change, yet these interventions operate within information environments that are actively shaped by commercial interests. Industry narratives can normalize harmful behaviours, shift responsibility onto individuals, and weaken support for effective public health policies. As a result, even well-designed interventions may have limited impact if they are undermined by persuasive, well-resourced communication strategies that influence how risks and solutions are understood.

Despite growing recognition of these strategies, public health responses remain fragmented and often reactive. There is a need for practical, communication-focused approaches that enable timely and coordinated responses. This paper addresses this gap by proposing a tactical health communication playbook.

## Current state of knowledge

A substantial body of research has documented how industries that profit from harmful products, including tobacco, alcohol, ultra-processed foods, and fossil fuels, use highly developed strategies to shape science, policy, and public opinion. These approaches, often described as corporate or industry ‘playbooks’, rely on a set of recurring tactics that transcend sectors and contexts (Reed *et al*. 2021, Moodie 2022, Thacker 2022). These include framing harms as matters of individual responsibility, promoting products as innovative or beneficial, selectively using or distorting scientific evidence, and obscuring risks through complexity or abstraction. Together, these strategies contribute to a persistent imbalance, enabling corporate narratives to overshadow evidence-based public health communication.

A key feature of these playbooks is the consistency and coordination with which they are deployed. Industry actors maintain message discipline across organizational levels, from corporate leadership to frontline communication, ensuring that core narratives are repeated, adapted, and reinforced across multiple platforms. By contrast, public health responses are often fragmented, spanning a diverse set of actors including non-governmental organizations, academic institutions, and public agencies, each with differing mandates, resources, and timelines. This asymmetry creates opportunities for industries to exploit divisions and delay or dilute regulatory action.

Existing scholarship has made important progress in conceptualizing these dynamics. Frameworks addressing the commercial determinants of health and corporate political activity have identified a wide range of strategies, including the manufacture of doubt, the management of conflicts of interest, coalition-building, and the strategic deployment of scientific and public relations tools (Reed *et al*. 2021, Moodie 2022, Thacker 2022). Building on this work, Lacy-Nichols *et al*. (2022) have proposed a ‘public health playbook’ to challenge corporate influence, identifying key strategic priorities such as capacity building, monitoring, coalition formation, and debunking misleading industry arguments.

However, despite these advances, current responses remain largely oriented towards high-level strategy rather than practical implementation. Much of the literature focuses on describing industry practices or outlining broad institutional responses, with less emphasis on how public health actors can engage effectively in real-time communication. As a result, responses to industry messaging are often reactive, piecemeal, and slower to emerge than the narratives they seek to counter. This gap is particularly evident in rapidly evolving media environments, where simplified and emotionally resonant messages can spread quickly and shape public perceptions before evidence-based responses are mobilized.

While existing frameworks identify important strategic priorities, they offer less guidance on how public health actors should respond in real time to evolving industry narratives. This creates a gap between strategic intent and practical communication action.

## Conceptual approach

This paper advances a conceptual and practice-oriented framework for public health communication, focusing on the tactical level of response. It argues that while strategic frameworks are necessary, they are insufficient without clear, actionable tactics that can be deployed rapidly to counter evolving industry narratives in real time.

We used an iterative, deliberative process drawing on the authors’ expertise in public health policy, commercial determinants of health, and strategic communication. Their work spans sectors including tobacco, alcohol, ultra-processed foods, and fossil fuels and includes framing and narrative analysis.

The starting point was the framework proposed by Lacy-Nichols and colleagues, which provided the conceptual foundation. We then held structured, iterative discussions to identify industry practices that could be countered through narrative and communication strategies, informed by case studies, advocacy experience, and available empirical evidence.

We aimed to distil tactics that met three criteria: applicability across domains, relevance to recurring industry frames, and practical usability. Ideas were generated independently and refined collectively, with disagreements resolved by prioritizing clarity and feasibility. The resulting six tactics represent those considered most effective and broadly applicable, complementing existing strategic frameworks while remaining distinct.

**Figure 1:**
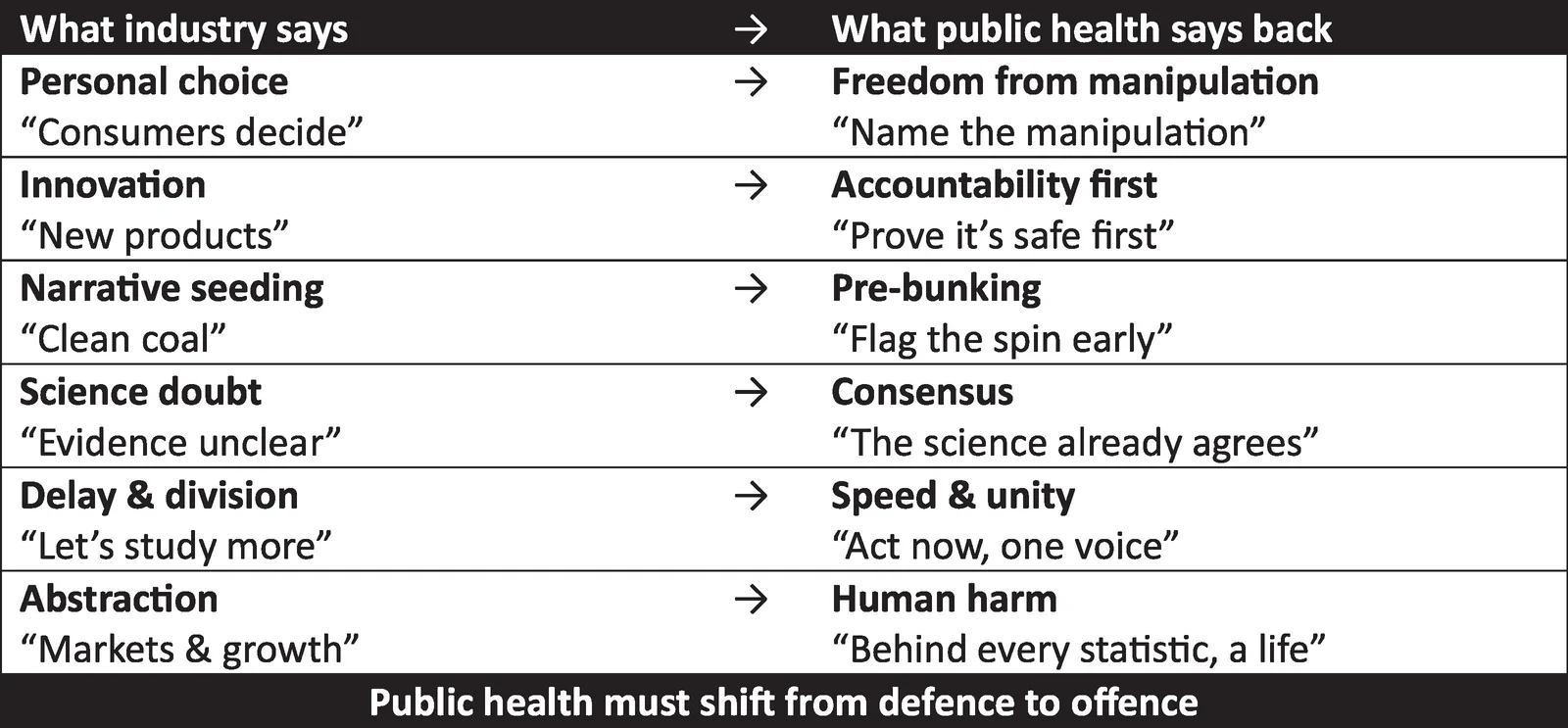
Two playbooks.

We identified six core tactics applicable across public health domains (Fig. 1). First, reframing ‘freedom of choice’ as ‘freedom from manipulation’ highlights how marketing and misinformation undermine autonomy. Second, emphasizing accountability challenges industry claims of innovation, recognizing that progress without responsibility can amount to exploitation. Third, pre-emptive prebunking anticipates and counters misleading narratives through early, evidence-based communication. Fourth, amplifying scientific consensus reduces manufactured doubt by emphasizing agreement and trusted voices. Fifth, speed and unity enable rapid, coordinated responses that prevent industry dominance of the narrative. Finally, humanizing harms replaces abstract debate with compelling stories and visuals that make impacts visible and relatable. We consider each in turn.

### Tactic 1. Countering industry freedom narratives with a focus on freedom from manipulation and protecting youth

Industry actors often present a unified voice in policy debates, framing harmful consumption as a matter of ‘personal choice.’ This suggests individuals act freely and bear full responsibility, while obscuring the roles of marketing, misinformation, and addiction. Public health should reframe this as ‘freedom from manipulation’, recognizing that genuine choice requires accurate information and protection from distorted decision-making.

Across sectors, industries undermine these conditions: tobacco companies conceal addiction while promoting independence; alcohol brands emphasize ‘responsible drinking’ while targeting young people; food companies market ultra-processed products as affordable while exploiting vulnerable groups; and fossil fuel firms link consumption to freedom while downplaying harms. Reframing the debate shifts focus from individual blame to structural accountability, emphasizing that real freedom depends on transparency, regulation, and protection from corporate influence.

This approach is especially important for children and young people, who are highly vulnerable to marketing that exploits social norms, peer influence, and digital environments. Industries deliberately target these groups to establish lifelong consumption patterns through influencer marketing, gamified advertising, and sponsorship of cultural and sporting events. Examples include flavoured e-cigarettes promoted on social media, youth-oriented alcohol marketing, and pervasive online advertising for ultra-processed foods. Framing the issue as protecting young people from manipulation highlights the ethical case for stronger regulation, such as marketing restrictions, age controls, and greater transparency, while positioning these measures as essential to autonomy rather than paternalism.

However, education alone is insufficient. Focusing solely on individual behaviour risks shifting responsibility away from structural drivers, and restrictive measures can sometimes backfire by increasing product appeal. Effective strategies must therefore combine education with systemic interventions, including marketing controls, pricing policies, and transparency measures, to reduce exposure and reshape harmful environments.

### Tactic 2. Countering industry innovation with a focus on accountability

Health-damaging industries routinely invoke ‘innovation’ to legitimize new products while downplaying their risks. Public health must respond by shifting the focus from novelty to accountability: claims of progress should be supported by independent evidence, transparent reporting, and robust regulation.

Across sectors, products presented as breakthroughs often carry unresolved harms. Tobacco companies promote heated tobacco products and e-cigarettes as technological advances despite evidence of youth uptake and dual use (Banks *et al*. 2026). Food manufacturers market ‘plant-based’ ultra-processed products as healthy alternatives, while alcohol companies promote ‘low-calorie’ or ‘functional’ drinks under a wellness framing. These cases illustrate a consistent pattern: innovation is used to signal safety or benefit without meeting the evidentiary standards required to justify such claims.

Public health should therefore apply a precautionary approach, insisting that safety and population-level impact are established before widespread adoption. In this framing, innovation without accountability is not progress but the externalization of risk.

Food manufacturers tout ‘plant-based’ ultra-processed snacks as healthy alternatives, while alcohol companies promote ‘low-calorie’ or ‘functional’ beverages under the guise of wellness. Fossil fuel firms brand carbon capture as a climate solution even as they continue to produce large-scale emissions (Gunderson *et al*. 2020). These examples underscore the need for scepticism and rigorous evaluation. Public health must apply the precautionary principle, requiring that any innovation undergo independent assessment, transparent reporting, and robust regulatory oversight before being deemed beneficial. Innovation without accountability is exploitation; health protection, not corporate branding, must remain the ultimate goal.

### Tactic 3. Countering industry narrative seeding with pre-emptive pre-bunking

Industry narratives about innovation are rarely neutral; they are strategically introduced early to shape how products are understood before scrutiny intensifies. Terms such as ‘harm reduction’, ‘plant-based’, or ‘low-calorie’ are deployed to frame products as beneficial and to pre-empt regulation.

For example, fossil fuel companies have long used phrases such as ‘clean coal’ and ‘energy independence’ to pre-empt concerns about environmental harm while continuing large-scale emissions (Gunderson *et al*. 2020). The sugary drinks industry promotes slogans such as ‘hydration and energy’ ahead of discussions on sugar taxes, positioning its products as vital for active lifestyles. In these ways, industries roll out simplified narratives that link products to health, sustainability, or modern lifestyles to establish favourable assumptions before evidence enters the debate.

To counter this, public health must act with equal speed and foresight. A health playbook should prioritize early, evidence-based communication, anticipate likely industry frames, and clearly explain why they are misleading before they take hold. This pre-emptive approach draws on inoculation theory: forewarning people and exposing them to weakened versions of misleading arguments builds resistance, much like a vaccine (Lewandowsky and van der Linden 2021). Evidence supports this strategy: a meta-analysis of 42 studies (*N* = 42 530) shows that ‘prebunking’ improves the ability to detect misinformation (Lu *et al*. 2023), while a large YouTube experiment (*N* = 22 632) demonstrates that such messages can work at scale (Roozenbeek *et al*. 2022). By equipping audiences in advance, inoculation complements, rather than replaces, later debunking. For example, public health agencies can pre-empt claims that ‘energy drinks’ are beneficial by highlighting their links to obesity and cardiovascular risk, or challenge terms like ‘clean coal’ by emphasizing ongoing health harms. In short, success depends on framing the debate first, not correcting it later.

When misinformation emerges, speed and clarity are critical. Industries rely on simple, repeatable claims, such as ‘moderate drinking is healthy’ or ‘e-cigarettes are safe’, that quickly become entrenched if unchallenged. Public health responses must therefore be equally rapid, accessible, and compelling, supported by ready-to-use materials such as fact sheets, infographics, and social media content. For instance, responses to alcohol industry claims can highlight evidence that no level of consumption is safe (Anderson *et al*. 2023), while the widely circulated assertion that e-cigarettes are ‘95% safer than cigarettes’ can be dismantled by explaining its flawed origins (McKee and Capewell 2015). Similarly, claims that fortified cereals are healthy should be countered by explaining that added vitamins do not offset high sugar content. Public health must not only anticipate misleading narratives but also respond to them quickly and decisively when they arise.

### Tactic 4. Countering ‘Muddying Waters’ strategies with consensus amplification

Industries have long exploited uncertainty to delay regulation and weaken public confidence. By cherry-picking favourable studies, funding research designed to confuse, and amplifying minor disagreements, they manufacture doubt (Oreskes and Conway 2010). Tobacco companies refined this approach, even developing ‘Good Epidemiology Practice’ as a pseudo-standard to discredit legitimate evidence linking smoking to disease (Ong and Glantz 2001), later extending similar tactics to other sectors, including the alcohol sector (McCambridge *et al*. 2019). Today, these strategies are widely used across industries, from fossil fuels to ultra-processed foods.

Public health must respond on two fronts. First, it should emphasize clear, authoritative statements of scientific consensus from trusted bodies. For example, WHO declarations on *trans* fats and IPCC assessments linking fossil fuels to health impacts help counter industry narratives by showing that the weight of evidence supports action. Research demonstrates that perceived consensus acts as a ‘gateway belief’: correcting misperceptions about scientific agreement shifts beliefs and increases support for policy (van der Linden *et al*. 2015). These effects have been replicated at scale and confirmed in meta-analyses (Rode *et al*. 2025) and extended from climate change to issues such as COVID-19 (Kerr and van der Linden 2022).

Second, public health must also explain uncertainty where it exists. Uncertainty is a core feature of science, reflecting transparency rather than weakness. Communicating this clearly helps audiences understand that uncertainty does not justify inaction and prevents its misuse to undermine regulation. In short, an effective response combines clarity about consensus with honesty about uncertainty, presenting both as strengths rather than vulnerabilities.

### Tactic 5. Countering delay, divide, and self-regulation strategies by speed, unity, and enforcement

As outlined above, public health must be equipped to counter industry playbooks with speed, clarity, and professionalism. Central to this is a practical communications toolkit: while the tactics provide direction, they require tools that enable rapid, credible responses.

Industries dominate media narratives through polished messaging, influencer campaigns, and emotionally appealing imagery. Public health must match this pace and sophistication. This includes ready-to-use templates for op-eds, social media, and press releases that can be quickly adapted to emerging issues. However, single messages have limited impact; these tools are most effective when they reinforce consistent frames over time, shaping norms rather than winning isolated exchanges. For example, when soda companies frame sugar taxes as ‘unfair’, pre-prepared responses can highlight evidence from countries such as Mexico, where such policies have reduced sugary drink purchases and are projected to yield long-term health benefits (Basto-Abreu *et al*. 2019). Similarly, simple graphics can counter vaping narratives by illustrating trends in youth uptake.

Credible voices are equally important. Industries often rely on paid experts or front groups to legitimize their claims. Public health should therefore maintain a network of independent, media-trained experts across epidemiology, policy, and behavioural science, ready to provide authoritative commentary. For example, when fossil fuel companies promote ‘clean coal’, experts can clearly explain its health and environmental impacts.

Finally, evidence must be communicated in ways that resonate. Industries exploit the limited appeal of dense scientific text, favouring simple and emotionally engaging visuals. Public health should do the same, using infographics, maps, and compelling imagery to make evidence accessible. Campaigns such as the WHO’s anti-tobacco messaging show how powerful visual storytelling can shift perceptions and cut through competing narratives.

### Tactic 6. Countering abstraction and dehumanization by revealing real human consequences to humanize the issue

Industries often rely on abstract ideas such as ‘freedom’ or ‘choice’ while obscuring the real human consequences of their products. Public health can counter this by amplifying personal stories that make harm visible and relatable. Accounts of individuals living with chronic obstructive pulmonary disease after smoking, or young people hospitalized with vaping-related lung injury, bring risks into sharp focus. Similarly, stories of families affected by alcohol-related crashes or children developing obesity and diabetes due to aggressive food marketing shift the debate from corporate rights to human impact.

These harms are not evenly distributed. Industries systematically target vulnerable communities, deepening health inequities: tobacco marketing has long focused on low-income and minority populations, fast-food outlets cluster in areas with limited access to fresh food, and fossil fuel extraction disproportionately exposes nearby communities to pollution and respiratory disease. Highlighting these patterns reframes the issue as a matter of social justice, showing that harm results from deliberate strategies rather than chance.

Numbers alone rarely persuade. As seen during the pandemic, even simple concepts can be difficult to communicate, and complex graphics often fail to engage. Effective communication, therefore, combines clear visuals with human stories. Evidence supports this approach: pictorial warnings outperform text-only messages in attracting attention and strengthening emotional responses and intentions to quit smoking (Noar *et al*. 2016), and they increase actual quit attempts (Brewer *et al*. 2016). The mechanism is straightforward; vivid, personal depictions of harm capture attention and prompt emotional engagement that abstract statistics cannot. Similarly, climate–health campaigns that show families affected by floods or children with asthma from air pollution cut through industry narratives by reminding audiences that behind every statistic is a human life.

## Implications for health promotion

### Research implications

This framework has several implications for health promotion research. It highlights the need to move beyond descriptive analyses of commercial determinants towards actionable, real-time communication strategies. Staying ahead of industry tactics is essential. Companies that profit from harmful products continually adapt their messaging to preserve legitimacy and pre-empt regulation.

Digital platforms intensify this challenge by spreading misleading narratives at speed. Alcohol brands, for example, use influencers to normalize drinking among young audiences (Strowger *et al*. 2024), while energy drink companies target gamers with claims of enhanced performance. Public health must therefore act early. Social listening can identify emerging trends, such as hashtags linked to vaping or sugary drinks, before they become entrenched, enabling timely and targeted counter-messaging.

Industry strategies are transnational and highly coordinated. Tobacco companies replicate marketing tactics across regions, soda manufacturers lobby against sugar taxes worldwide, and fossil fuel firms fund think tanks in multiple countries to shape climate policy. Public health must respond with the same level of coordination. Useful models already exist: the WHO tobacco industry interference database allows governments to share intelligence on lobbying and marketing practices (World Health Organization 2026); the STOP (Stopping Tobacco Organisations and Products) network tracks global tobacco industry tactics (STOP 2026); the Global Alcohol Policy Alliance (2026) supports cross-national collaboration on alcohol policy; and climate-health coalitions such as the Global Climate and Health Alliance (2026) bring organizations together to counter fossil-fuel narratives.

Health protection offers several lessons here. It works through surveillance, early warning, intelligence sharing, and coordinated response rather than waiting until a threat is fully established. The same principles can be applied to harmful industry narratives. Just as outbreak control depends on detecting signals early, sharing information quickly, and acting across borders, public health communication should identify emerging industry frames, assess their spread, and coordinate rapid counter-messaging. This shifts the task from *ad hoc* rebuttal to a more systematic model of prevention and preparedness.

The health playbook should therefore formalize these approaches into a global early-warning system that combines media monitoring, social listening, and shared intelligence platforms. Such a system would enable rapid detection of new industry narratives, comparison of tactics across countries, and coordinated responses before harmful frames become entrenched. In this sense, public health can learn from health protection not only the value of vigilance but also the importance of organized, cross-border infrastructure for anticipating and containing threats.

There is also a need to evaluate the effectiveness of communication tactics in real-world settings. While evidence supports approaches such as prebunking and consensus messaging, further research should assess how these strategies perform across different populations, media platforms, and policy contexts. In particular, studies should examine how communication interacts with underlying structural and political factors, including regulation, enforcement, and industry influence.

Finally, future research should prioritize interdisciplinary approaches that integrate insights from public health, political science, communication studies, and behavioural science. Such integration is essential to understanding not only how industry narratives operate but how they can be effectively countered in complex and contested information environments.

### Policy implications

Effective communication must be supported by enabling policy environments. Strong policies are essential to limit industry influence, but transparency alone is not sufficient. Measures such as bans on industry-funded research in academic journals set important precedents, yet disclosure by itself does not prevent conflicts of interest or undue influence. Transparency is necessary but must be accompanied by accountability and enforcement.

The experience of tobacco control illustrates the potential for progress. The WHO Framework Convention on Tobacco Control Article 5.3 was a landmark step in protecting public health policy from industry interference. A growing body of evidence suggests that similar safeguards are needed across sectors, including alcohol, ultra-processed foods, and fossil fuels, which collectively contribute to a substantial burden of preventable disease and death. A WHO Europe (2024) report estimates that just four industries, tobacco, alcohol, ultra-processed foods, and fossil fuels, cause 2.7 million deaths in the European Region every year. Nor are industry playbooks limited to these sectors. Infant formula companies frame aggressive marketing as support for mothers, gambling firms promote ‘responsible gaming’ while resisting regulation, and pesticide manufacturers present their products as essential for food security despite growing evidence of ecological and health harms.

Policies should therefore focus on strengthening conflict-of-interest regulations, ensuring full disclosure of direct and indirect funding, and preventing industry-funded research from unduly influencing policy. Measures such as journal bans on tobacco-funded research set an important precedent, yet disclosure by itself cannot prevent conflicts of interest or undue influence. Transparency is not equivalent to accountability: an actor can be fully transparent about harmful practices and still face no meaningful consequences. The health playbook should promote enforceable standards that prevent industry-funded science from passing as independent evidence and back them with systems to monitor compliance and sanction breaches.

Industries often hide financial relationships through front groups or indirect funding (Steele *et al*. 2019). Tobacco companies, for example, have channelled funds through bodies such as the Foundation for a Smoke-Free World (Daube *et al*. 2017), while soda companies sponsor community programmes that shift attention away from calorie intake and towards physical inactivity (Steele *et al*. 2019). Mandatory disclosure of all funding sources, including indirect links, is therefore essential. One model is the U.S. Open Payments database, which tracks financial ties between physicians and pharmaceutical companies (U.S. Centers for Medicare & Medicaid Services 2026). Evidence suggests that such registries improve transparency and can influence behaviour, although scrutiny comes mainly from journalists rather than the public (Kanter 2019). Similar systems are needed across sectors such as food, alcohol, and fossil fuels. Disclosure will not eliminate conflicts of interest, but it is a necessary first step.

Crucially, policy design must address both the strength of rules and their implementation. Policies without enforcement are ineffective. Two problems must be distinguished: rules that are weak by design and full of loopholes, and rules that are adequate on paper but poorly enforced. Industries exploit both. Examples include marketing energy drinks to minors despite age restrictions and promoting ‘low-tar’ cigarettes as safer products (Pollay and Dewhirst 2002). To prevent this, regulators need meaningful penalties, including fines, licence revocation, and publication bans for undisclosed conflicts of interest. Enforcement matters: policies such as the EU *trans* fat ban were effective because they were supported by compliance monitoring and sanctions.

By contrast, weak enforcement is common and costly. Voluntary alcohol marketing codes have repeatedly failed to prevent advertising aimed at young people, while poor enforcement of age restrictions has allowed e-cigarette use to spread among adolescents in many countries. In the food sector, self-regulatory pledges on junk food marketing to children have also proved ineffective, especially online. These examples show that without strong compliance systems and sanctions, industries will continue to delay, dilute, or evade regulation. The health playbook must therefore pair policy with enforcement strong enough to protect public health.

### Practice implications

Industry playbooks succeed through speed, simplicity, and repetition, allowing harmful narratives to spread faster than evidence-based messages. These tactics sit within a longer-term strategy: industries invest heavily in public relations, lobbying, and framing to shape norms and policy over decades. Public health practitioners cannot afford to remain reactive.

It is important to note that although we refer to a ‘public health’ playbook, public health is not a single, unified actor and lacks the unifying profit motive that can animate and integrate disparate actors. Public health has a community that includes NGO staff, Boards, public institutions (e.g. QUANGOs), and international organizations, and these groups often operate with different priorities and at different speeds.

If the public health community can rally around shared playbook messaging, it may help overcome longstanding internal fragmentation that can lead to delays and incoherence. Health-harming industries tend to have highly aligned messaging across staff, leadership, and governance structures. In contrast, public health is often slower to reach internal agreement within a single entity, due to a mismatch between staff and the Board. If public health organizations can forge a preliminary agreement around a shared playbook, it may empower staff who are ready to act but wait on boards and governance structures that can be slow to adapt, refocus, and realign priorities across organizations.

Across these sectors, industries have become adept at presenting harm as ‘choice’, ‘innovation’, or ‘responsibility’, while using coordinated strategies to shape science, policy, and public opinion. The health playbook offers a way to counter this systematically, not only by rebutting misinformation, but by anticipating and disrupting industry narratives before they take hold. This requires reframing debates, using evidence strategically, humanizing harm, strengthening policy and regulation, monitoring industry tactics, and building a robust communications toolkit. In short, public health must move from defence to offence, adopting a proactive and coordinated approach that matches the speed, sophistication, and persistence of industry strategies. Taking this forward requires action on multiple fronts:

- **Institutional and governance adoption:** Convene a cross-sector coalition (umbrella bodies such as the membership-led public health coalitions, such as HEAL, European Heart Network, Smoke Free Partnership and Eurocare, alongside disease-specific NGOs) to agree on a shared playbook messaging guide, and have WHO, national public health agencies, academic institutions, and the European Public Health Association adopt it in their advocacy and policy work.
- **Capacity building:** Prioritize training for board members of public health NGOs, who are key decision-making actors and are often less equipped than staff for rapid communication responses; also train public health professionals and NGO staff in rapid response communication, media engagement, and social listening. Training should extend beyond communication skills to encompass the political and rhetorical competencies that shape policy debates (framing, narrative, and the strategic use of evidence), embedded in public health curricula and NGO media programmes.
- **Global collaboration:** Build on existing networks (the WHO tobacco-industry interference database, STOP, the Global Alcohol Policy Alliance, and the Global Climate and Health Alliance) to create a shared rapid-response hub that pools intelligence and harmonizes counter-narratives across borders. Regional and national alliances, such as the NCA Alliance and Fresh Balance (2026), offer additional models of sustained cross-sector collaboration that deserve wider recognition.
- **Investment in tools:** Jointly fund a shared repository of ready-to-use templates, visuals, and fact sheets, hosted by a membership-led umbrella organization rather than an intergovernmental body and maintained for rapid deployment by members.
- **Policy advocacy:** Researchers, journals, and regulators should jointly press for transparency, enforceable regulations, and publication standards that keep industry-funded science out of health policy.
- **Critical self-reflection:** Look inward as well as out. Public health should ask which of its own practices, framings, and metaphors are ineffective or inadvertently serve industry interests, identify the barriers to changing them, and be willing to stop doing what no longer works.
- **Meet the moment:** Work within today’s polarized and resource-constrained politics; harness emerging tools such as artificial intelligence as a force for good; and centre the voices of young people and others who carry the heaviest burden of industry harm yet are least often heard.

## Conclusion

The health playbook is not a static document. It is a living strategy. Its success depends on commitment, including endorsement by the governance structures and board members of public health NGOs, as well as their funders, coordination, and continuous adaptation to emerging industry tactics. Public health must shift from defence to offence, ensuring that truth, equity, and health protection prevail over corporate profit. Ultimately, this is a call for more than new tactics: it asks public health to reskill and to rethink the commercial drivers of harm and what it will take to promote health in the twenty-first century.

## Data Availability

Not applicable.
